# Raphe glucose-sensing serotonergic neurons stimulate KNDy neurons to enhance LH pulses via 5HT2CR: rat and goat studies

**DOI:** 10.1038/s41598-024-58470-4

**Published:** 2024-05-03

**Authors:** Sho Nakamura, Takuya Sasaki, Yoshihisa Uenoyama, Naoko Inoue, Marina Nakanishi, Koki Yamada, Ai Morishima, Reika Suzumura, Yuri Kitagawa, Yasuhiro Morita, Satoshi Ohkura, Hiroko Tsukamura

**Affiliations:** 1 Laboratory of Animal Production Science, Graduate School of Bioagricultural Sciences, Nagoya University, Togo-cho, Aichi 470-0151 Japan; 2 Laboratory of Animal Reproduction, Graduate School of Bioagricultural Sciences, Nagoya University, Nagoya, Aichi 464-8601 Japan

**Keywords:** Neuroscience, Reproductive biology, Neurophysiology

## Abstract

Dysfunction of central serotonergic neurons is known to cause depressive disorders in humans, who often show reproductive and/or glucose metabolism disorders. This study examined whether dorsal raphe (DR) serotonergic neurons sense high glucose availability to upregulate reproductive function via activating hypothalamic arcuate (ARC) kisspeptin neurons (= KNDy neurons), a dominant stimulator of gonadotropin-releasing hormone (GnRH)/gonadotropin pulses, using female rats and goats. RNA-seq and histological analysis revealed that stimulatory serotonin-2C receptor (5HT2CR) was mainly expressed in the KNDy neurons in female rats. The serotonergic reuptake inhibitor administration into the mediobasal hypothalamus (MBH), including the ARC, significantly blocked glucoprivic suppression of luteinizing hormone (LH) pulses and hyperglycemia induced by intravenous 2-deoxy-D-glucose (2DG) administration in female rats. A local infusion of glucose into the DR significantly increased in vivo serotonin release in the MBH and partly restored LH pulses and hyperglycemia in the 2DG-treated female rats. Furthermore, central administration of serotonin or a 5HT2CR agonist immediately evoked GnRH pulse generator activity, and central 5HT2CR antagonism blocked the serotonin-induced facilitation of GnRH pulse generator activity in ovariectomized goats. These results suggest that DR serotonergic neurons sense high glucose availability to reduce gluconeogenesis and upregulate reproductive function by activating GnRH/LH pulse generator activity in mammals.

## Introduction

Dysfunction of central serotonergic neurons is known to cause depressive disorders in humans, who often show reproductive and/or glucose metabolism disorders^1,2^. The reproductive and glucose metabolism disorders associated with depression may be due to central disturbance in the function of serotonergic signaling, as depression is mainly caused by the dysfunction of serotonergic neurons in the brain; thus, serotonin reuptake inhibitors are often used as a therapeutic treatment in depression patients. Therefore, uncovering the role of serotonergic neurons in controlling reproduction and glucose metabolism may contribute to new therapeutic controls for reproductive disorders in patients suffering from depression.

The raphe nuclei in the brainstem, such as the dorsal raphe (DR) or median raphe nucleus, are the major source of serotonergic neurons^3^, and serotonergic neuronal fibers widely project to several brain regions, including the mediobasal hypothalamus (MBH), amygdala, and cortex in male rats^4^. Importantly, our previous study showed that serotonergic neurons in the DR and median raphe nucleus coexpress pancreatic-type glucokinase, a restriction enzyme for glucose metabolism, in female rats^5^. Furthermore, our in vitro study showed that intracellular Ca^2+^ levels in serotonergic neurons taken from the raphe obscurus nucleus of female rats increased in response to high extracellular glucose concentrations in the medium^6^. These findings suggest that raphe serotonergic neurons may sense an increase in glucose availability to regulate glucose metabolism, but it is unclear whether serotonergic neurons sense and mediate energy status to regulate reproductive function.

Kisspeptin (encoded by *Kiss1* gene) neurons are known to be a key regulator of mammalian reproduction because loss-of-function mutations in the kisspeptin receptor (also known as GPR54) gene result in hypogonadotropic hypogonadism in humans, and deletion of *Kiss1* or *GPR54* in mice and rats causes infertility in both sexes^7–10^. Kisspeptin neurons located in the hypothalamic arcuate nucleus (ARC), coexpressing neurokinin B (NKB) and dynorphin A (Dyn), thus also called KNDy neurons, serve as a source of the gonadotropin-releasing hormone (GnRH) pulse generator in mammals, including primates^11^, ruminants^12–14^, and rodents^9,15,16^. Indeed, our previous study showed that pulsatile LH release and folliculogenesis were restored in global *Kiss1* KO rats by an injection of adeno-associated virus vectors containing *Kiss1* cDNA into ARC *Tac3* (NKB gene)-expressing cells to rescue KNDy neurons^17^. Furthermore, our previous studies showed that rhythmic increases in multiple unit activity (MUA) volleys, which are synchronized with circulating LH pulses, were detected only when the electrodes were placed in a cluster of ARC kisspeptin neurons in male and female goats^12,13^. Furthermore, a central single administration of NKB immediately evoked MUA volley and then increased the MUA volley frequency taken from the ARC of OVX goats, indicating that recording MUA volleys in goat ARC is a powerful tool to monitor the activity of KNDy neurons, that is the GnRH pulse generator.

It has been well known that malnutrition suppresses reproductive function in several mammalian species^10^, such as rodents^18,19^, ruminants^20,21^ and primates^22^, including humans^23^. Importantly, GnRH/LH pulse generator activity is suppressed during lowered energetic conditions because fasting profoundly decreased the frequency of the LH pulse in female rats^24^ and sheep^21^ and prolonged the MUA volley intervals recorded in the ARC of OVX goats treated with luteal phase levels of 17β-estradiol (E2)^20^. Among the nutrients, lowered glucose availability is a key factor in inhibiting reproduction, because a peripheral administration of 2-deoxy-D-glucose (2DG), an inhibitor of glucose utilization, caused inhibition of estrous cycle in hamsters^18^ and rats^25^, and peripheral/central 2DG-induced glucoprivation suppressed LH pulses in female rats^26,27^, goats^28^, and sheep^29^. Furthermore, insulin-induced hypoglycemia suppressed GnRH pulse generator activities and pulsatile LH release in female goats^28^and rhesus monkeys^30^. These findings suggest that glucose availability plays a key role in maintaining GnRH/LH pulse generation and then reproductive function in mammals, including ruminants and monogastric animals. The involvement of μ-, κ-, and δ-opioid receptor signaling in malnutrition-induced suppression of ARC KNDy neurons was indicated in female rats^26,31–33^, while no report is available which shows a role of serotonergic neurons in the regulation of GnRH pulse generator activity in response to status and changes in energy/glucose availability in any species, including rodents and ruminants. Thus, it is worth clarifying the role of central serotonergic signaling in the regulation of GnRH/LH pulse generation in rodents (as a monogastric animal model including humans) and ruminants (as a model for livestock, such as cattle).

A recent RNA-seq analysis using *Kiss1*-Cre-activated ZsGreen-expressing cells of female mice showed that ARC kisspeptin neurons express mRNAs encoding several types of serotonin receptors, such as inhibitory *Htr5a* and excitatory *Htr2a*, *Htr4*, and *Htr7*^34^. This finding suggests that kisspeptin neurons could be a putative target of serotonin to regulate reproduction and energy metabolism. On the other hand, the physiological role of serotonin in controlling GnRH/LH pulses is under debate because serotonin exerts both facilitatory and inhibitory effects on LH pulses in ovariectomized (OVX) rats^35^, consistent with the expression of both inhibitory and excitatory serotonin receptors in mouse ARC kisspeptin neurons^34^.

The present study, therefore, was intended to clarify the hypothesis that DR serotonergic neurons sense high glucose availability and positively regulate reproductive function via excitatory serotonin receptors expressed in ARC kisspeptin neurons in mammals using female rats and goats, as models of monogastric animals and ruminants. To this end, we first performed RNA-seq analysis using tdTomato-positive kisspeptin neurons taken from *Kiss1*-tdTomato heterozygous female rats to investigate the types of serotonergic receptors. Since the expression of the serotonin-2C receptor (5HT2CR) gene (encoded by *Htr2c*), a Gq-coupled excitatory receptor, was evident by RNA-seq analysis of rat ARC kisspeptin neurons, we performed double in situ hybridization to verify the coexpression of 5HT2CR in kisspeptin neurons in the ARC and anteroventral periventricular nucleus (AVPV), another major nucleus for kisspeptin neurons, in female rats. Next, we investigated whether the administration of fluoxetine, a serotonergic reuptake inhibitor, into the MBH, including the ARC, blocks the suppression of LH pulses and an increase in blood glucose levels in female rats treated with intravenous (iv) administration of 2DG as an experimental model of malnutrition. Furthermore, we investigated whether a direct infusion of glucose into the DR increases in vivo serotonin release in the MBH using microdialysis and restores LH pulses in iv-2DG-treated female rats. Finally, using an electrophysiological technique recording MUA volley in ARC kisspeptin neurons of female goats, we examined whether a central administration of serotonin and a 5HT2CR agonist stimulates GnRH pulse generator activity and LH release and whether the central antagonism of 5HT2CR signaling blocks the serotonin-induced stimulation of GnRH pulse generator activity and LH release in female goats.

## Results

### *Htr2c* expression in ARC *Kiss1* neurons determined by RNA-seq analysis and double in situ hybridization

**Figure 1:**
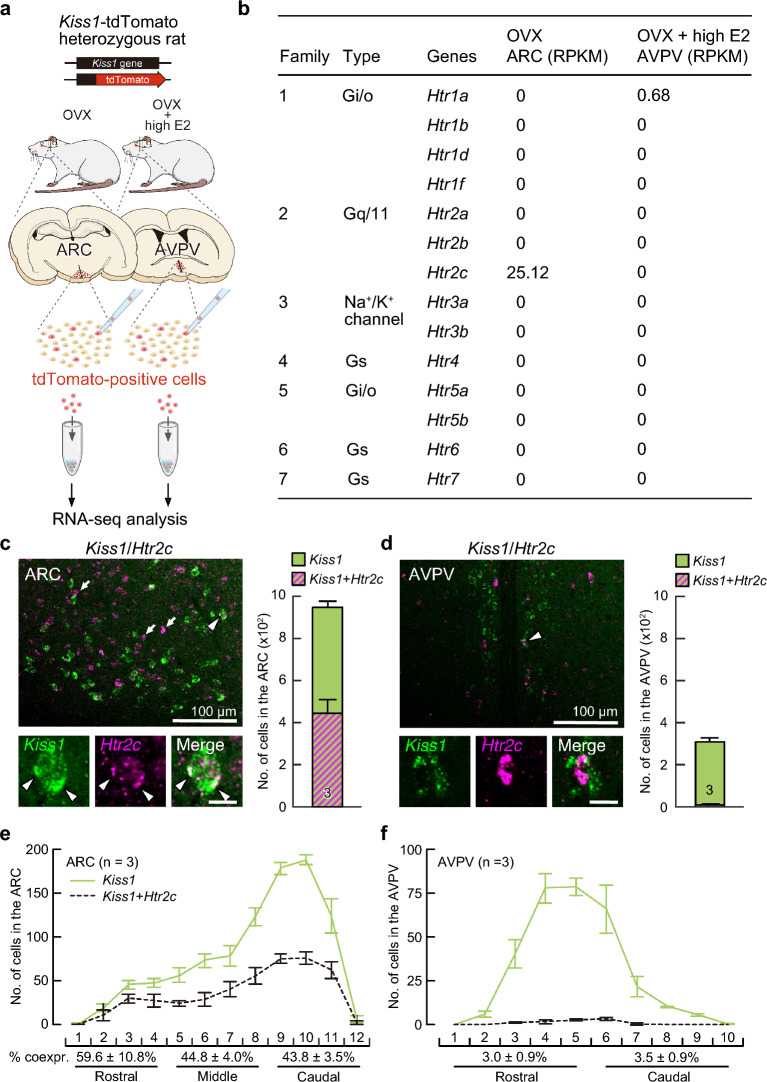
Gene expression profiles of serotonin receptors analyzed by RNA-seq in tdTomato-positive kisspeptin neurons taken from the arcuate nucleus (ARC) or anteroventral periventricular nucleus (AVPV) and double in situ hybridization for kisspeptin (*Kiss1*) and serotonin-2C receptor (*Htr2c*) mRNA expression in the ARC and AVPV in female rats. (**a**) Schematic illustration of the procedure of RNA-seq analysis using *Kiss1*-tdTomato heterozygous female rats. tdTomato-positive ARC kisspeptin neurons were taken from ovariectomized (OVX) *Kiss1*-tdTomato heterozygous rats, and AVPV kisspeptin neurons were taken from OVX *Kiss1*-tdTomato heterozygous rats treated with proestrous levels of estradiol-17β (OVX + high E2). (**b**) Gene expression profiles of serotonin receptors analyzed by RNA-seq with tdTomato-positive ARC or AVPV *Kiss1*-tdTomato cells taken from OVX or OVX + high E2 *Kiss1*-tdTomato heterozygous female rats, respectively. The values were normalized by the reads per kilobase million (RPKM) mapped reads for each mRNA. (**c**, **d**) *Kiss1*-expressing (green) and *Htr2c*-expressing cells (magenta) in the ARC (**c**) and AVPV (**d**) of representative OVX wild-type rats treated with negative feedback levels of E2 (OVX + low E2). The magnified images indicate *Kiss1*- and *Htr2c*-coexpressing cells indicated by an arrowhead. Arrows indicate *Kiss1*-negative *Htr2c*-expressing cells. Scale bars, 100 µm. Scale bars in inset, 10 µm. The number of cells expressing *Kiss1* alone (green column) or both *Kiss1* and *Htr2c* (green and magenta striped column) was quantified in the ARC (**c**) and AVPV (**d**) of OVX + low E2 rats. Values are means ± standard errors of the means (SEMs). The number in each column indicates the number of animals used. (**e**, **f**) The distribution of *Kiss1*-positive cells (green solid line) and *Kiss1*-positive cells coexpressing *Htr2c* (dashed line) throughout the ARC (**e**) and AVPV (**f**). The ARC was divided into the rostral (from 1.8 to 2.6 mm posterior to the bregma), middle (from 2.6 to 3.4 mm posterior to the bregma), and caudal (from 3.4 to 4.2 mm posterior to the bregma) parts. The AVPV was divided into the rostral (from 0.0 to 0.5 mm anterior to the bregma) and caudal (from 0.0 to 0.5 mm posterior to the bregma) parts. The percentage of *Kiss1*-expressing cells coexpressing *Htr2c* was calculated in the rostral, middle, and caudal parts of the ARC (n = 3, e) and the rostral and caudal parts of the AVPV (n = 3, f). Values are means ± SEMs.

RNA-seq analysis of ARC and AVPV kisspeptin neurons isolated from *Kiss1*-tdTomato heterozygous female rats (Fig. 1a) revealed that *Htr2c* was largely expressed in ARC kisspeptin neurons, but gene expression of other serotonergic receptors, such as *Htr1a*, *Htr1b*, *Htr1d*, *Htr1f*, *Htr2a*, *Htr2b*, *Htr3a*, *Htr3b*, *Htr4*, *Htr5a*, *Htr5b*, *Htr6*, and *Htr7*, was undetectable in the ARC kisspeptin neurons of OVX *Kiss1*-tdTomato rats (Fig. 1b). Few serotonergic receptor gene expression was detected regardless of type in the AVPV kisspeptin neurons in OVX + high E2 *Kiss1*-tdTomato rats.

Double in situ hybridization for *Kiss1* and *Htr2c* mRNA showed that some *Kiss1*-expressing cells coexpressed *Htr2c* mRNA and some *Kiss1*-negative *Htr2c*-expressing cells were also present in the ARC of OVX + low E2 rats (Fig. 1c). Quantitative analysis revealed that almost half (46.7 ± 4.3%, n = 3, Fig. 1c) of ARC *Kiss1*-expressing cells coexpressed *Htr2c*. On the other hand, little *Htr2c* was found in the AVPV *Kiss1*-expressing cells (Fig. 1d), and the ratio of AVPV *Kiss1*-expressing cells coexpressing *Htr2c* was 3.3 ± 0.4% (n = 3, Fig. 1d). The percentage of *Kiss1*-expressing cells coexpressing *Htr2c* was 59.6 ± 10.8, 44.8 ± 4.0, and 43.8 ± 3.5% in the rostral, middle, and caudal parts of the ARC, respectively (n = 3, Fig. 1e). The percentage of *Kiss1*-expressing cells coexpressing *Htr2c* was 3.0 ± 0.9 and 3.5 ± 0.9% in the rostral and caudal parts of the AVPV, respectively (n = 3, Fig. 1f). No significant difference was found in the coexpression ratio among the parts of the ARC or AVPV.

### An increase in serotonergic tone by an injection of fluoxetine, a serotonin reuptake inhibitor, into the MBH blocked glucoprivic suppression of the LH pulse and the glucoprivic increase in plasma glucose concentrations in female rats

**Figure 2:**
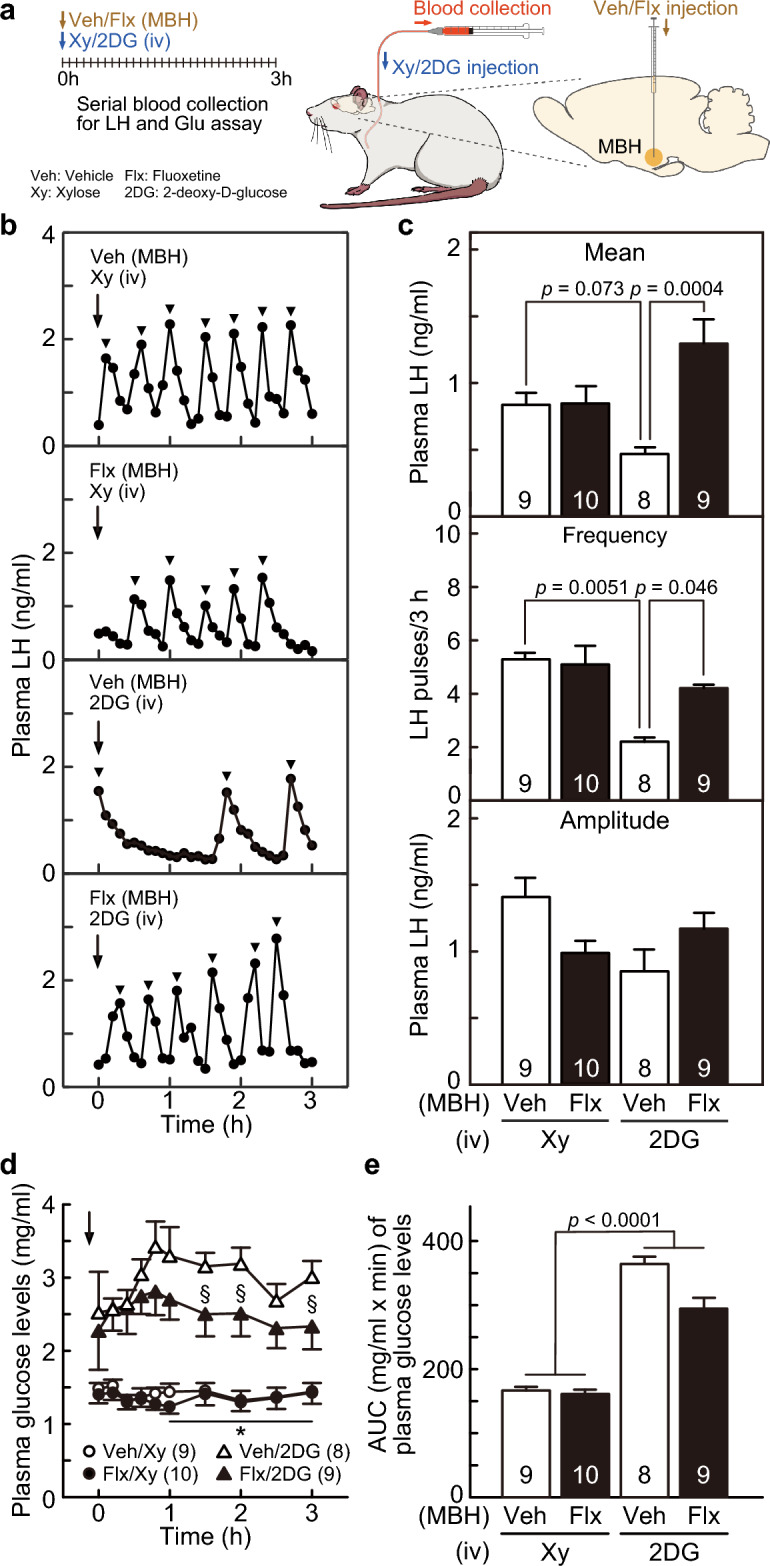
Administration of fluoxetine (Flx), a serotonin reuptake inhibitor, into the mediobasal hypothalamus (MBH) blocked glucoprivic suppression of luteinizing hormone (LH) pulses and gluconeogenesis induced by an intravenous (iv) injection of 2-deoxy-D-glucose (2DG) in OVX + low E2 rats. (**a**) Schematic illustration of the experimental procedure. The rats were treated with MBH injection of vehicle (Veh) or Flx and then with iv injection of xylose (Xy) or 2DG at the first blood sampling of 3-h sampling period. (**b**) Plasma LH profiles in representative OVX + low E2 rats, which were treated with MBH injection of Veh or Flx and then iv injection of Xy or 2DG. Arrowheads indicate the peaks of LH pulses identified by the PULSAR computer program. (**c**) The mean LH concentrations and the frequency and amplitude of LH pulses in each group. Significant differences were found in the mean LH concentration between the 2DG (iv)-Flx (MBH) and 2DG (iv)-Veh (MBH) groups (*p* = 0.0004) and in the frequency of LH pulses between the 2DG (iv)-Veh (MBH) and Xy (iv)-Veh (MBH) groups (*p* = 0.0051) and the 2DG (iv)-Flx (MBH) and 2DG (iv)-Veh (MBH) groups (*p* = 0.046). (**d**) Changes in the mean plasma glucose levels in OVX + low E2 rats treated with MBH injection of Veh or Flx and iv injection of Xy or 2DG. *Significant differences (*p* < 0.005) between the Xy (iv)-Veh (MBH) and 2DG (iv)-Veh (MBH) and between the Xy (iv)-Flx (MBH) and 2DG (iv)-Flx (MBH) groups. §Significant differences (*p* < 0.05) between the 2DG (iv)-Veh (MBH) and 2DG (iv)-Flx (MBH) groups. In addition, the plasma glucose levels tended to be lower (*p* = 0.094 at 1 h after 2DG administration) in the 2DG (iv)-Flx (MBH) group than in the 2DG (iv)-Veh (MBH) group. (**e**) The area under the curve (AUC) of plasma glucose levels for the last 2-h sampling period. A significant difference (*p* < 0.0001) was found in the AUC of plasma glucose levels between the Xy- and 2DG-treated groups. Values are means ± SEMs. The numbers in each column indicate the number of animals used.

We investigated the effect of the administration of fluoxetine, a serotonin reuptake inhibitor, into the MBH on 2DG-induced suppression of LH pulses and gluconeogenesis using glucoprivic malnutritional model rats receiving iv 2DG injection (Fig. 2a). Figure 2b shows the plasma LH profiles of representative OVX + low E2 rats receiving an MBH injection of fluoxetine or vehicle (dimethyl sulfoxide (DMSO)) just before an iv injection of 2DG or xylose. Apparent LH pulses were observed throughout the 3-h sampling period in xylose (iv)-vehicle (MBH) control rats, whereas LH pulses in the 2DG (iv)-vehicle (MBH) rats were suppressed compared with the control group. Notably, MBH fluoxetine treatment blocked the suppressive effect of 2DG on pulsatile LH release and restored apparent LH pulses in the 2DG (iv)-fluoxetine (MBH) group. Two-way ANOVA revealed that there was a significant main effect of fluoxetine (MBH) on the mean LH concentrations [F (1,32) = 10.57, *p* = 0.0027], a significant main effect of 2DG (iv) on the frequency of LH pulses [F (1,32) = 10.83, *p* = 0.0024], and an interaction of main effects (2DG × fluoxetine treatments) on the mean LH concentrations [F (1,32) = 10.39, *p* = 0.0029] and the frequency of LH pulses [F (1,32) = 3.41, *p* = 0.074]. Specifically, the mean LH concentration tended to be lower (*p* = 0.073, Fig. 2c) in 2DG (iv)-vehicle (MBH)-treated rats than in xylose (iv)-vehicle (MBH)-treated rats, whereas the mean LH concentration was significantly higher (*p* = 0.0004, Fig. 2c) in 2DG (iv)-fluoxetine (MBH)-treated rats than in 2DG (iv)-vehicle (MBH)-treated rats. In addition, the frequency of LH pulses was significantly lower (*p* = 0.0051, Fig. 2C) in 2DG (iv)-vehicle (MBH)-treated rats than in xylose (iv)-vehicle (MBH)-treated rats, whereas the frequency of LH pulses in 2DG (iv)-fluoxetine (MBH)-treated rats was significantly higher (*p* = 0.046, Fig. 2c) than that in 2DG (iv)-vehicle (MBH)-treated rats. There were no significant main effects or interactions on the amplitude of LH pulses.

Figure 2d shows changes in plasma glucose concentrations for the 3-h blood sampling period in OVX + low E2 rats receiving an iv injection of 2DG or xylose and an MBH injection of fluoxetine or vehicle. Plasma glucose levels were immediately increased by iv 2DG administration and were maintained at high levels in the 2DG (iv)-vehicle (MBH) group, whereas the levels were significantly lowered by fluoxetine treatment into the MBH in 2DG (iv)-treated female rats (Fig. 2d). In contrast, the plasma glucose levels were maintained at low levels in the xylose (iv)-vehicle (MBH) and xylose (iv)-fluoxetine (MBH) groups throughout the sampling period. Three-way repeated-measures ANOVA revealed that there were significant main effects [2DG, F (1, 32) = 54.19, *p* < 0.0001, and time, F (9, 128) = 3.16, *p* = 0.016] and a significant interaction of main effects (2DG × time) on the plasma glucose concentrations during the last 2-h sampling period [F (9, 128) = 4.38, *p* = 0.002]. Specifically, the plasma glucose levels in the 2DG (iv)-vehicle (MBH) and 2DG (iv)-fluoxetine (MBH) groups were significantly higher than those in the xylose (iv)-vehicle (MBH) and xylose (iv)-fluoxetine (MBH) groups, respectively (*, *p* < 0.005, from 1 to 3 h after 2DG administration; Fig. 2d). In addition, the plasma glucose levels in the 2DG (iv)-fluoxetine (MBH) group tended to be lower at 1 h (*p* = 0.094) and were significantly lower at 1.5 (§, *p* = 0.029), 2 (§, *p* = 0.029), and 3 h (§, *p* = 0.038) after iv 2DG administration than those in the 2DG (iv)-vehicle (MBH) group (Fig. 2d). The area under the curve (AUC) of plasma glucose levels during the last 2-h sampling period in the 2DG (iv) groups was significantly higher than that in the xylose (iv) groups [(F (1, 32) = 54.66, *p* < 0.0001, two-way ANOVA; Fig. 2e].

### Local glucose administration into the DR increased serotonin release in the MBH, blocked glucoprivic LH pulse suppression, and partly blocked the glucoprivic increase in plasma glucose concentrations in female rats

**Figure 3:**
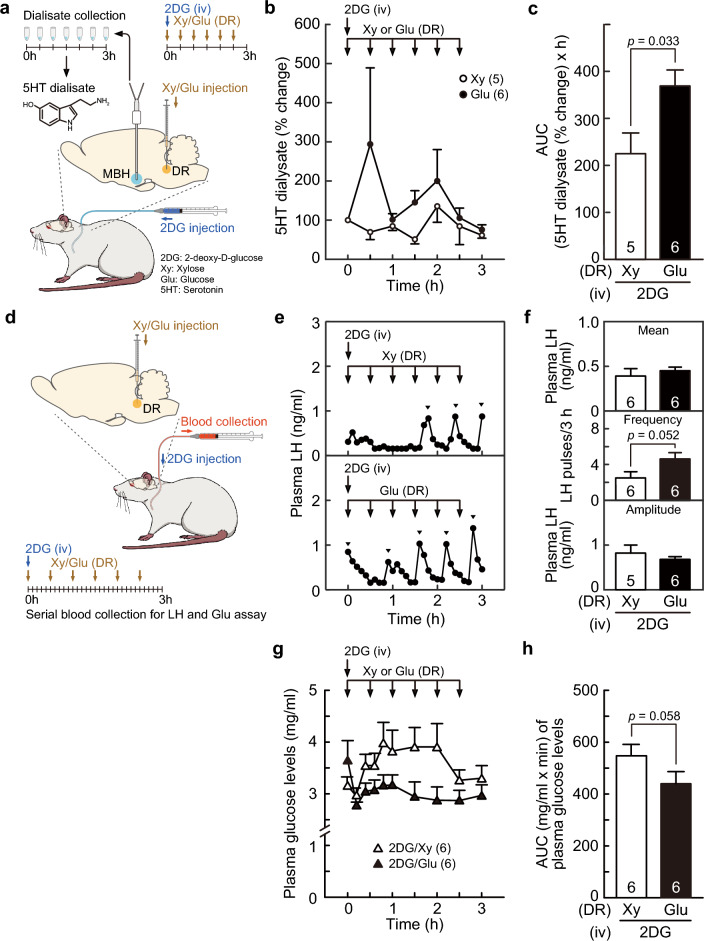
Local infusion of glucose into the dorsal raphe (DR) increased serotonin (5HT) release in the MBH and blocked 2DG-induced suppression of LH pulses in female rats. (**a**) Schematic illustration of the microdialysis procedure for determining extracellular 5HT levels in the MBH of OVX + low E2 rats. Xy or Glu was administered into the DR at 30-min intervals, 2DG was administered just after the first Xy or Glu injection, and microdialysates were collected every 30 min from the MBH. (**b**) Percent changes in the 5HT contents in MBH microdialysates in Xy- or Glu-treated female rats bearing iv 2DG. Arrows indicate the timing of repeated DR injections of Xy or Glu. The MBH 5HT levels tended to be higher (*p* = 0.066) in DR Glu-treated rats than in DR Xy-treated controls. (**c**) The area under the curve (AUC) of the 5HT levels in microdialysates (from 0.5 to 3 h) in DR Glu-treated rats was significantly higher than that in DR Xy-treated controls (*p* = 0.033). (**d**) Schematic illustration of the experimental procedure for determining effect of DR injection of Xy or Glu on iv 2DG-indued LH suppression in OVX + low E2 rats. (**e**) Plasma LH profiles in representative DR Glu or Xy-treated glucoprived rats. Arrowheads indicate the peaks of LH pulses identified by the PULSAR computer program. (**f**) The mean LH concentrations and the frequency and amplitude of LH pulses in each group. The frequency of LH pulses tended to be higher (*p* = 0.052) in DR glucose-treated glucoprived rats than in DR Xy-treated glucoprived controls. (**g**) The changes in the mean plasma glucose levels in DR Glu or Xy-treated glucoprived rats. The plasma glucose levels tended to be lower (*p* = 0.066 and 0.074 at 1.5 and 2 h after 2DG administration) in DR Glu-treated glucoprived rats than in DR Xy-treated glucoprived controls. (**h**) The AUC of plasma glucose levels for the last 2-h sampling period tended to be lower in DR Glu-treated glucoprived rats than in DR Xy-treated glucoprived controls. Values are means ± SEMs. The numbers in each column indicate the number of animals used.

We investigated the effect of local administration of glucose into the DR on serotonin release in the MBH of OVX + low E2 rats receiving peripheral 2DG injection (Fig. 3a). Analysis of percent changes in serotonin content in microdialysate collected from the MBH showed that the MBH serotonin levels increased after the injections of glucose into the DR in glucose-treated rats, whereas the levels failed to show an obvious change after the DR xylose injection in DR xylose-treated rats throughout the 3-h sampling period (Fig. 3b). Two-way repeated-measures ANOVA revealed that the MBH serotonin levels tended to be higher in DR glucose-treated rats than in xylose-treated controls [F (1, 9) = 4.38, *p* = 0.066, Fig. 3b]. Importantly, the AUC of percent changes in MBH serotonin levels in 2DG (iv)-glucose (DR) rats was significantly higher than that in 2DG (iv)-xylose (DR) rats (*p* = 0.033, Student’s *t-*test, Fig. 3c).

Next, we investigated the effect of local administration of glucose into the DR on 2DG-induced suppression of LH pulses and gluconeogenesis in female rats receiving peripheral 2DG injection (Fig. 3d). Figure 3e shows plasma LH profiles in representative OVX + low E2 rats receiving iv injection of 2DG with the repeated injection of glucose or xylose into the DR. In the 2DG (iv)-xylose (DR) control rats, LH pulses were suppressed after 2DG treatment, whereas frequent LH pulses were recovered by glucose injection into the DR in 2DG (iv)-glucose (DR) rats (Fig. 3e). Statistical analysis revealed that the frequency of LH pulses in 2DG (iv)-glucose (DR) rats tended to be higher than that in 2DG (iv)-xylose (DR) control rats (*p* = 0.052, Student’s *t*-test, Fig. 3f). There was no significant difference in the mean LH concentrations and amplitude of LH pulses between the 2DG (iv)-glucose (DR) and 2DG (iv)-xylose (DR) groups (Fig. 3f).

Figure 3g shows changes in plasma glucose concentrations for the 3-h blood sampling period in OVX + low E2 rats receiving an iv injection of 2DG and a repeated DR injection of glucose or xylose. Plasma glucose levels were maintained at higher levels in the 2DG (iv)-xylose (MBH) group than in the 2DG (iv)-glucose (MBH) group. Two-way repeated-measures ANOVA revealed that a main effect [glucose; F (1, 10) = 4.56, *p* = 0.058] on the plasma glucose concentrations during the last 2-h sampling period tended to be significant. Specifically, the plasma glucose concentration of the 2DG (iv)-glucose (DR) group tended to be lower than that of the 2DG (iv)-xylose (DR) group at 1.5 (*p* = 0.066) and 2 h (*p* = 0.074) after iv 2DG administration (Fig. 3g). The AUC of plasma glucose concentrations during the last 2-h sampling period in 2DG (iv)-glucose (DR) rats tended to be lower than that in 2DG (iv)-xylose (DR) control rats (*p* = 0.058, Student’s *t*-test, Fig. 3h).

### Serotonin-5HT2CR signaling activates GnRH pulse generator activity and increases LH release in OVX goats

**Figure 4:**
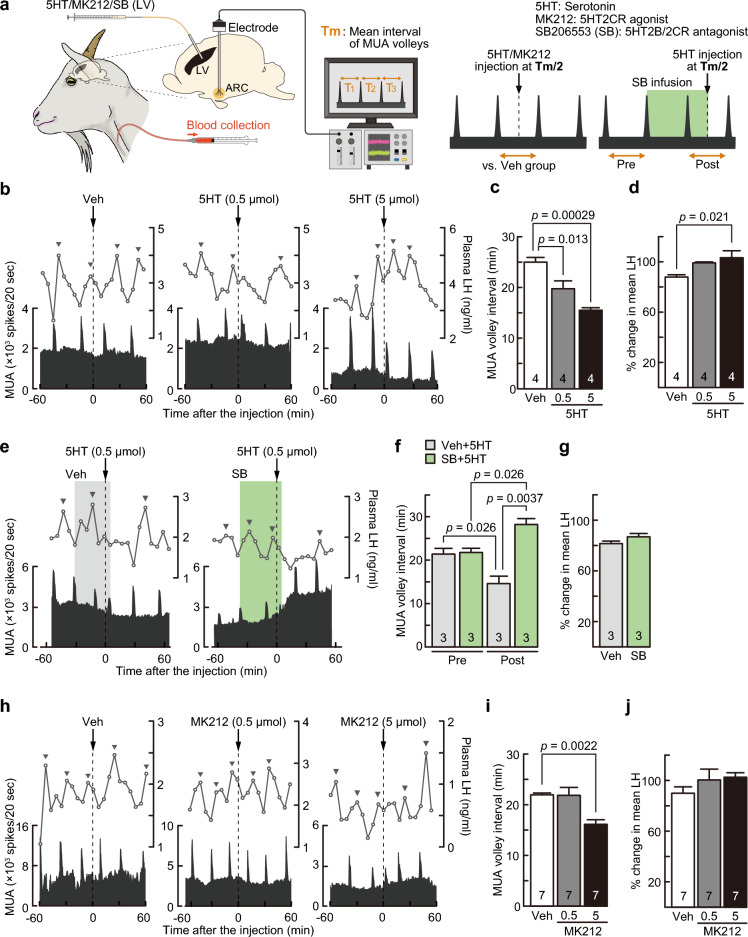
Central administration of serotonin (5HT) and serotonin-2C receptor (5HT2CR) agonist stimulated GnRH pulse generator activity, and 5HT2CR antagonism blocked the 5HT-induced stimulation of GnRH pulse generator activity recorded by multiple unit activity (MUA) in the ARC of OVX goats. (**a**) Schematic illustration of the experimental procedure for determining the effects of lateral ventricle (LV) administration of 5HT and 5HT2CR agonist and antagonist on MUA volley in goats. 5HT and MK212, a 5HT2CR agonist, were injected into the LV at a half-time of the mean interval of MUA volleys (Tm/2) determined by the 5-h prerecording of MUA. SB206553 (SB), a 5HT2CR antagonist, was infused into the LV immediately after the second MUA volley until the 5HT injection at Tm/2 after the third MUA volley. (**b**) Profiles of MUA volleys and LH pulses in representative goats treated with 5HT or Veh (arrows). Arrow heads indicate the peaks of LH pulses identified by the PULSAR computer program. (**c**) The mean MUA volley interval of OVX goats after central 5HT (0.5 or 5 µmol) administration was significantly (*p* < 0.05) shortened compared with that of Veh-treated control goats. (**d**) Percent change in mean LH levels of OVX goats after central 5HT (5 µmol) administration significantly (*p* < 0.05) increased compared with that of Veh-treated controls. (**e**) Profiles of MUA volleys and LH pulses of representative goats treated with LV infusion of SB (green) or Veh (gray) and LV injection of 5HT (arrows). (**f**) The mean MUA volley intervals at the postinfusion period were significantly (*p* = 0.0026) shortened by 5HT injection in the Veh-treated group (gray), whereas the interval at the postinfusion period significantly (*p* = 0.0026) elongated in the SB-treated group (green). In addition, significant differences were found in the MUA volley interval between the SB + 5HT and Veh + 5HT groups during the postinfusion period (*p* = 0.0037). (**g**) The percent change in mean LH levels in OVX goats treated with LV infusion of SB (green) or Veh (gray). (**h**) Profiles of MUA volleys and LH pulse of representative OVX goats treated with LV administration of MK212 (0.5 or 5 µmol) or Veh (arrows). (**i**) The mean MUA volley interval of OVX goats treated with central administration of MK212 (5 µmol) was significantly shorter than that of Veh-treated controls (*p* = 0.0022). (**j**) The percent change in mean LH levels in OVX goats treated with LV administration of MK212 (0.5 or 5 µmol) or Veh. Values are means ± SEMs. The numbers in each column indicate the number of animals used. Arrowheads indicate the peaks of LH pulses identified by the PULSAR computer program.

Finally, we examined the effects of serotonergic signaling on GnRH pulse generator activity using the electrophysiological MUA recording technique in the ARC of OVX goats (Fig. 4a). The central administration of serotonin (0.5 or 5 µmol) immediately evoked an MUA volley, as shown in representative goats (Fig. 4b), resulting in significantly shorter MUA volley intervals in both the 0.5 (*p* = 0.013, one-way ANOVA followed by Dunnett’s test) and 5 µmol (*p* = 0.00029) serotonin-administered groups compared with the vehicle-treated control group (Fig. 4c). In addition, 5 µmol serotonin administration increased plasma LH concentration, resulting in a significantly higher percent change in mean LH levels (*p* = 0.021, one-way ANOVA followed by Dunnett’s test) in the serotonin-administered group (5 µmol) compared with that in the vehicle-treated controls (Fig. 4d).

The central infusion of SB206553, a selective 5HT2CR antagonist, blocked the facilitatory effect of serotonin on the MUA volley (Fig. 4e). Two-way repeated-measures ANOVA revealed that there was a significant main effect [SB206553; F (1, 4) = 110.82, *p* = 0.0005] and interaction of main effects (SB206553 × time) [F (1, 4) = 30.90,* p* = 0.0051] on the MUA volley interval in OVX goats. Specifically, in the vehicle-serotonin group, the MUA volley interval at the postinfusion period was significantly shortened by serotonin injection compared with the preinfusion period (*p* = 0.026, Fig. 4f). Importantly, the MUA volley interval at the postinfusion period was significantly elongated in SB206553-serotonin-injected goats compared with the preinfusion period in the SB206553-serotonin group (*p* = 0.026, Fig. 4f) and the postinfusion period of the vehicle-serotonin group (*p* = 0.0037, Fig. 4f). There was no significant difference in the percent change in mean LH levels between groups (Fig. 4g). Furthermore, the central administration of MK212 (5 µmol), a 5HT2CR agonist, immediately induced an MUA volley (Fig. 4h), resulting in an MUA volley interval in MK212 (5 µmol)-treated goats that was significantly shorter than that in the vehicle-treated control group (*p* = 0.0022, one-way ANOVA followed by Dunnett’s test, Fig. 4i). A lower dose (0.5 µmol) of MK212 failed to affect the occurrence of MUA volley in OVX goats. There was no significant difference in the percent change in mean LH levels between groups (Fig. 4j).

## Discussion

The present study demonstrated that DR serotonergic neurons can sense glucose availability to upregulate reproductive function by activating GnRH/LH pulse generation in mammals; this is because glucoprivic inhibition of LH pulses was blocked by an increase in hypothalamic serotonergic tone by the MBH injection of fluoxetine, a serotonergic reuptake inhibitor, or local infusion of glucose into the DR in female rats. Furthermore, the present study showed that the expression of 5HT2CR, an excitatory serotonin receptor, was evident in approximately half of ARC kisspeptin neurons, which is known as the GnRH pulse generator^36–40^, in female rats, and central administration of serotonin or a 5HT2CR agonist immediately evoked MUA volley, which is an indicator of GnRH pulse generator activity in female goats. Furthermore, central administration of a 5HT2CR antagonist blocked the serotonin-induced occurrence of MUA volley and elongated the MUA volley interval in female goats. These findings suggest that serotonin-5HT2CR signaling may enhance GnRH pulse generator activity via direct or indirect stimulation of ARC KNDy neurons through 5HT2CR expressed in the neurons or other neighboring cells; this notion is largely consistent with a recent in vitro study showing that serotonin treatment elicits the firing frequency of some ARC kisspeptin neurons in female mouse brain slices^34^. In addition, our previous in vitro study showed that rat raphe serotonergic neurons were activated by an increase in extracellular glucose levels^6^. Taken together, these findings suggest that raphe serotonergic neurons can sense glucose availability to upregulate GnRH pulse generation, resulting in the facilitation of GnRH/LH pulses in mammals. To the best of our knowledge, this is the first report showing the glucose-sensing role of serotonergic neurons and 5HT2CR signaling in direct stimulation of GnRH pulse generator activity. Thus, the current findings contribute to the development of new therapeutic controls for reproductive disorders in patients suffering from depression, whose central serotonergic function is largely suppressed. Further studies are required to determine whether serotonergic signaling could block LH pulse suppression in feed-restricted models for further understanding of the physiological role of central serotonergic signaling in regulation of reproductive function.

The present histological analysis revealed that approximately half of the rat ARC KNDy neurons expressed stimulatory 5HT2CR mRNA, which is consistent with the current rat and previous mouse RNA-seq analysis results^34^; this suggests that the 5HT2CR-expressing KNDy neurons could be a direct action site of serotoninergic inputs from the DR to control GnRH/LH pulses. Importantly, ARC KNDy neurons interact with each other via NKB-NKB receptor and Dyn-κ-opioid receptor signaling and gap junctions in rodents^26,41,42^, goats^43^, and sheep^44,45^. Thus, serotonergic inputs received by half of the KNDy neurons may upregulate the synchronized activity of the cluster of KNDy neurons. Indeed, the current electrophysiological study showed that a central administration of serotonin or the 5HT2CR agonist “immediately” evoked an MUA volley taken from a cluster of the ARC kisspeptin neurons of female goats. In addition to the direct serotonergic inputs to KNDy neurons, it is also possible that ARC KNDy neurons receive indirect facilitatory serotonergic inputs via non-KNDy ARC neurons because *Htr2c*-positive signals were found outside of the *Kiss1*-expressing cells in the ARC of female rats. Indeed, it was reported that 80% of ARC α-melanocyte-stimulating hormone (α-MSH)-immunopositive cells express the 5HT2CR gene in male rats^46^ and that approximately 40% of ARC proopiomelanocortin (POMC, precursor of α-MSH)-immunopositive neurons express 5HT2CR in mice^47^. Adrenocorticotropic hormone (ACTH, also encoded by the *Pomc* gene)-immunopositive cells receive inputs from serotoninergic fibers terminating in the ARC of male rats^48^. Moreover, administration of the selective agonist for melanocortin-4 receptor (MC4R), an α-MSH receptor, into the lateral ventricle (LV) induced LH elevation in male rats, and α-MSH-immunoreactive fibers were identified in close apposition to ARC kisspeptin-immunoreactive cells in female rats^49^. In goats, LV administration of MC3/4R agonist or antagonist shortened or prolonged the MUA volley interval, respectively^50^. Approximately 70% of ARC kisspeptin neurons express either MC3R or MC4R in male sheep^51^. Furthermore, a monosynaptic trac-tracing study in mouse brain showed that the greatest inputs to KNDy neurons originating from non-KNDy ARC neurons are POMC neurons in mice^52^. In ewe, POMC-immunoreactive fibers contact with approximately 30% of ARC kisspeptin neurons in OVX ewe^53^. Collectively, these findings suggest that α-MSH neurons receiving serotonergic inputs may partly mediate serotonin-induced stimulation of ARC KNDy neurons. Interestingly, it was reported that serotonin-1A receptor (5HT1AR), an inhibitory Gi/o protein-coupled receptor, is expressed in ARC neuropeptide Y (NPY)/agouti-related peptide (AgRP) neurons^54^, and ARC AgRP neurons are suggested to directly inhibit KNDy neuronal activity and suppress fertility in mice^55^. Indeed, approximately 70% of ARC kisspeptin neurons showed close contact with AgRP-immunoreactive terminals in male sheep^51^, as do 30% of ARC kisspeptin with NPY-immunoreactive fibers in OVX ewe^53^. Moreover, intracerebroventricular infusion of NPY largely inhibited the activity of GnRH pulse generator as indicated by MUA volley taken from the ARC of female goats^56^ and suppressed pulsatile LH secretion in OVX ewe^57^. These findings suggest that serotonergic inputs may also indirectly enhance KNDy neuronal activity by inhibiting NPY/AgRP neurons through inhibitory 5HT1AR expressed in the NPY/AgRP neurons in addition to the direct stimulation of KNDy neurons via 5HT2CR in rodents and ruminants.

It is likely that DR serotonergic neurons are at least partly involved in glucose metabolism in rats because the 2DG-induced increase in blood glucose levels was reversed by MBH administration of fluoxetine, and local administration of glucose into the DR partly lowered the 2DG-induced increase in plasma glucose levels in female rats. It was reported that male mice with a genetic mutation of 5HT2CR showed a higher blood glucose concentration than wild-type mice 2 h after a single intraperitoneal injection of glucose^58^, and POMC neuron-specific 5HT2CR gene deficiency caused hyperglycemia in mice^59^. Moreover, an intraperitoneal injection of a 5HT2CR agonist blocked an increase in blood glucose concentrations after intraperitoneal glucose injection in male mice with type 2 diabetes, and this effect of the 5HT2CR agonist on glucose downregulation requires the activation of downstream MC4R signaling because the effect of the 5HT2CR agonist was not detected in MC4R gene KO mice^60^. Taken together, the increase in serotonergic tone in the hypothalamus induced by the current MBH fluoxetine or DR glucose treatments may have activated ARC POMC neurons via stimulatory 5HT2CR and then blocked the glucoprivic increase in plasma glucose concentrations via the MC4R signaling pathway in female rats.

The present study showed that the central administration of serotonin or 5HT2CR agonist immediately evoked MUA volley taken from the ARC in female goats, suggesting that serotonergic neurons may transmit the information on glucose availability to enhance the GnRH pulse generator activity in ruminants, even though the volatile fatty acids produced through the ruminal fermentation are the major energy sources in ruminants^61^. In support of this notion, the GnRH pulse generator activity was suppressed by 2DG-induced glucoprivation or insulin-induced hypoglycemia in female goats because the intravenous infusion of 2DG or insulin prolonged the interval of MUA volleys as an indicator of GnRH pulse generator activity in OVX goats treated with luteal phase levels of E2^28^. Indeed, there is a temporal correlation between blood glucose concentrations and the activity of GnRH pulse generator, as higher glucose concentrations showed shorter MUA volley intervals in female goats^28^. Moreover, intracerebral insulin administrations inhibited pulsatile LH secretion in OVX ewe^62^. Taken together, these and current findings suggest that serotonergic signals may transmit the information of glucose availability to regulate GnRH/LH pulse generation and then reproductive function in ruminants.

The ependymocytes surrounding the fourth cerebral ventricle (4V) are likely to play a role in sensing low glucose availability^6^, because the 4V infusion of 2DG suppressed LH pulses in both sheep and rats^63,64^. Furthermore, our in vitro study showed that lowered extracellular glucose levels increased intracellular Ca^2+^ levels in the ependymocytes taken from the 4V of rats. Hindbrain noradrenergic neurons located in the vicinity of the 4V are suggested to transmit the glucoprivic signals to the paraventricular nucleus (PVN)^65^, resulting in an activation of PVN Dyn^26^ and/or corticotropin-releasing hormone neurons^66^ to suppress pulsatile LH secretion in rats. On the other hand, a high glucose availability condition can be sensed by the raphe serotonergic neurons as the current glucose infusion into the DR blocked glucoprivic suppression of LH pulse in female rats. This notion is consistent with our previous study showing that high extracellular glucose concentrations increased intracellular Ca^2+^ levels of serotonergic neurons taken from the raphe obscurus nucleus of rats^6^. Given that the serotonin-5HT2CR signaling upregulates GnRH pulse generator activity in goats and the signaling blocked glucoprivic suppression of LH pulses in rats in the present study, there is the mechanism that serotonergic neurons may enhance GnRH pulse generator activities in response to an increase in glucose availability in goats as in rats. Collectively, high and low glucose availability can be monitored by raphe serotonergic neurons and hindbrain ependymocytes, respectively, and the energetic information transmitted to the hypothalamus would be integrated by ARC KNDy neurons to control GnRH pulse generation in response to the nutritional changes in mammalian species.

**Figure 5:**
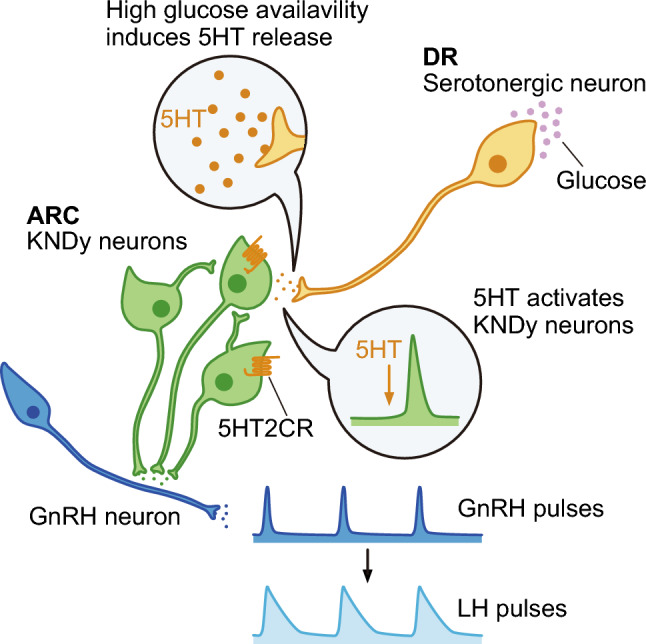
The schematic illustration shows the putative network between dorsal raphe (DR) serotonergic neurons and arcuate nucleus (ARC) KNDy neurons regulating GnRH pulse generation during high glucose availability. High glucose availability, which may be sensed by the DR serotonergic neurons, induces serotonin (5HT) release in the mediobasal hypothalamus including the ARC from the DR serotonergic neurons. The serotonergic signaling stimulates ARC KNDy neurons through 5HT2C receptors (5HT2CR) expressed in the neurons to facilitate GnRH/LH pulse generation.

In conclusion, the present study demonstrated that DR serotonergic neurons sense glucose availability to upregulate reproductive function via direct/indirect activation of GnRH/LH pulse generation in female rats. Specifically, the current findings suggest that serotonin-5HT2CR signaling may enhance GnRH pulse generator activity via direct or indirect stimulation of ARC KNDy neurons in rats and goats (Fig. 5). To the best of our knowledge, this is the first in vivo study showing the role of serotonergic neurons in sensing energy availability to enhance GnRH/gonadotropin pulses and mammalian reproduction. Therefore, this finding contributes to the establishment of a new therapeutic approach for reproductive disorders in depressed patients, the central serotonergic tone of which is largely suppressed.

## Methods

### Animals and treatments

Wild-type female Wistar-Imamichi (Institute for Animal Reproduction, Ibaraki, Japan) and *Kiss1*-tdTomato heterozygous rats (8–13 weeks old, 180–250 g)^9^ were used. Animals were fed CE-2 pellets (CLEA Japan) and water ad libitum under a 14 L:10 D (light on 5:00 am) photoperiod at 22 ± 2 °C. *Kiss1*-tdTomato heterozygous female rats, whose coding sequence for *Kiss1* gene was replaced with tdTomato gene, were subjected to RNA-seq analysis as described previously^67^. Wild-type female rats were used for in vivo and histological experiments to determine the role of DR serotonergic neurons in monitoring glucose availability to control pulsatile LH release and blood glucose levels.

Wild-type female rats with two consecutive estrous cycles were OVX and implanted with subcutaneous Silastic tubing (inner diameter, 1.57 mm; outer diameter, 3.18 mm; 25 mm in length; Dow Corning, Midland, MI, USA) containing E2 (Sigma‒Aldrich) dissolved in peanut oil (20 µg/mL, Sigma‒Aldrich) for 7 days to serve as OVX + low E2 rats. This E2 treatment was confirmed to mimic diestrous level of E2 to show negative feedback to LH pulses^24^ and the OVX + low E2 rats were used for glucoprivation model according to our previous study, showing that peripheral 2DG treatment (400 mg/kg BW) suppressed LH pulses in OVX + low E2 rats but not in OVX rats^27^. *Kiss1*-tdTomato heterozygous female rats with two consecutive estrous cycles were OVX, and some of them received a subcutaneous implant of Silastic tubing (inner diameter 1.0 mm; outer diameter 1.5 mm; length 20 mm) filled with crystalline E2 for 2 days, followed by 4 days of low E2 implantation in OVX rats to serve as a proestrous model, OVX + high E2 rats^68^. The surgical procedures were performed under ketamine (26.7 mg/kg)/xylazine (5.3 mg/kg) mixture and inhalant 1–2% isoflurane (Pfizer Japan) anesthesia.

Adult female Shiba goats (n = 14, 25–33 kg), a non-seasonal breeder^69^, were kept under the natural lighting condition and used for MUA recording. All goats were OVX more than 1 month prior to the onset of the MUA experiment. The OVX model was chosen for MUA recording, because the model is suitable to identify frequent MUA volleys and thus useful to examine the effects of some drugs on GnRH pulse generator activity in goats^70^. The goats were kept loosely tied to an individual stanchion under a 12 L:12 D (lights on 8:00 am) photoperiod at 23 °C and were fed a standard pelleted diet (Acemare; Nosan) and dry hay (Hey cube; Konki). Water and supplemental minerals (Salt lick; Kyoritsu Seiyaku) were available ad libitum.

All experimental procedures were approved by the Committee of Animal Experiments of the Graduate School of Bioagricultural Sciences, Nagoya University. This study was conducted in accordance with the Nagoya University Regulations on Animal Care and Use in Research and ARRIVE guidelines.

### Analysis of RNA-seq data from *Kiss1*-tdTomato heterozygous rats

Expression profiles of serotonergic receptor genes in ARC or AVPV kisspeptin neurons were analyzed using RNA-seq data reported previously^67^. Briefly, the ARC or AVPV tissues were dissected out from *Kiss1*-tdTomato heterozygous female rats under OVX (n = 2) or OVX + high E2 (n = 2) conditions, respectively, because tdTomato fluorescence expression was dependent on *Kiss1* expression and the tdTomato signals were only detectable in the ARC of OVX rats and the AVPV in OVX + high E2 model rats^9^. The ARC or AVPV tissues were minced and treated with papain and then gently dispersed by repetitive pipetting, and the tdTomato-positive cells were picked up under a fluorescent microscope by pipettes (inner diameter: 20–30 μm)^71^. Ten (for ARC) or 3 (for AVPV) tdTomato-positive cells were pooled into a polymerase chain reaction tube containing an RNase inhibitor (RNasin Plus; Promega, Madison, WI, USA). Gene expression levels were normalized by the reads per kilobase million mapped reads for each mRNA^67^.

### Serial blood sampling to determine the effects of MBH treatment with fluoxetine, a serotonin reuptake inhibitor, and DR administration of glucose on the suppression of LH pulses induced by peripheral 2DG injection

OVX + low E2 rats were used for glucoprivation model according to our previous study, showing that peripheral 2DG treatment (400 mg/kg BW) suppressed LH pulses in OVX + low E2 rats but not in OVX rats^27^. Seven days before the day of blood sampling, OVX + low E2 wild-type rats were stereotaxically implanted with stainless-steel guide cannulae (22G; Plastics ONE) into the MBH (n = 38) or DR (n = 12) according to the rat brain atlas^72^ as follows: at 2.3 mm posterior and 7.5 mm ventral to the bregma at 0.6 mm midline or at 3.8 mm posterior and 8.2 mm ventral to the bregma at 0.6 mm from midline for the MBH, and 7.8 mm posterior and 5.8 mm ventral to the bregma at the midline for the DR. The OVX + low E2 rats were inserted with a silicon cannula (0.5 mm i.d.; 1.0 mm o.d.; Shin-Etsu Polymer) into the right atrium through the jugular vein on the day before blood sampling. The free-moving conscious rats were administered fluoxetine hydrochloride (fluoxetine; Sigma‒Aldrich), a serotonin reuptake inhibitor, dissolved in DMSO (Sigma‒Aldrich) at 200 nmol/µL into the MBH at a flow rate of 0.5 µL/min for 1 min by microsyringe pump (EICOM) through an internal cannula (28G; Plastics ONE) immediately after the first blood sampling collected via the jugular vein cannula. D-glucose (glucose; Katayama Chemical Industries) or xylose (Katayama Chemical Industries), an indigestible sugar for rats, dissolved in artificial cerebrospinal fluid (aCSF; consisting of 147 mM NaCl, 4 mM KCl, 1 mM MgCl2, and 1.3 mM CaCl_2_) at 2 µmol/µL was administered into the DR at a flow rate of 0.1 µL/min for 3 min at the beginning of blood sampling and infused every 30 min at the same flow rate for 1 min during blood sampling. 2DG (400 mg/kg BW, Sigma‒Aldrich) dissolved at 200 mg/mL in saline or an equimolar hypertonic concentration of xylose (366 mg/kg BW) dissolved at 183 mg/mL in saline was intravenously (iv) injected, and 100 µL of blood was collected for 3 h at 6 min intervals. The doses of 2DG and xylose were chosen according to previous studies^26,31,33^, showing that LH pulses were significantly suppressed by iv 2DG treatment in OVX + low E2 rats. Plasma samples (50 µL) were obtained by immediate centrifugation and stored at − 20 °C until assayed for LH. Plasma glucose concentrations were measured in an additional volume (50 µL) of plasma samples obtained every 12 min during the first 1 h and every 30 min during the last 2 h of the blood sampling period, as previously described^73^. At the end of blood sampling, the animals were perfused with 10% formalin after the injection of the same volume of 3% brilliant blue solution using a microsyringe pump. The brain sections were prepared with a cryostat and stained with 0.2% thionin solution to verify the placement of the MBH and DR cannulae. The data taken from the animals showing the right placement of the MBH (36 out of 38 rats) and DR (12 out of 12 rats) cannulae were analyzed.

### Microdialysis for measurement of extracellular serotonin levels in the MBH after glucose infusion into the DR

OVX + low E2 rats were used for glucoprivation model according to our previous study, showing that peripheral 2DG treatment (400 mg/kg BW) suppressed LH pulses in OVX + low E2 rats but not in OVX rats^27^. At least 1 week before the microdialysis sampling, OVX + low E2 wild-type rats (n = 12) were stereotaxically implanted with guide cannulae for glucose infusion (C315G, Plastic One) and for microdialysis (AG-12, EICOM) in the DR and MBH, respectively. The coordinate of the tip of the guide cannula for microdialysis was 2.3 mm posterior and 9.7 mm ventral to the bregma at 0.6 mm from midline for the bregma. A microdialysis probe (A-I-12–02, EICOM) was connected to a 10 mL microsyringe with a swivel unit (SSU-20, EICOM). The probe was inserted in the MBH 1–2 h before the onset of glucose infusion into the DR. The MBH was then perfused continuously through the microdialysis probe with degassed aCSF using a microinjection pump at a flow rate of 1 µL/min. Microdialysates were collected every 30 min in vials containing 10 µL of 0.1 M acetic acid (Katayama Chemical Industries)-100 mg/L ethylenediaminetetraacetic acid (EDTA, Katayama Chemical Industries) on ice. Glucose or xylose for DR infusion was dissolved in aCSF at a concentration of 1.6 µmol/0.8 µL. The amount of serotonin in the microdialysates was measured by a high-performance liquid chromatography-electrochemical detector (HPLC-ECD; EICOM). On each sampling day, serotonin levels in the microdialysates were quantified according to the areas of the peaks detected by HPLC injected with the standard serotonin solution prepared from 1.5 pg/100 µL to 1000 pg/100 µL (serotonin creatinine sulfate complex; Sigma‒Aldrich). At the end of sampling, the animals were perfused with 10% formalin after the injection of 3% brilliant blue solution into the DR under anesthesia using the same protocol as that used for glucose infusion. The brain sections were prepared with a cryostat and stained with 0.2% thionin solution to verify the placement of the MBH and DR cannulae. The data taken from the animals (11 out of 12 rats) showing the right placement of the MBH and DR cannulae were analyzed.

### Double in situ hybridization for *Kiss1* and *Htr2c* in the ARC and AVPV

The OVX + low E2 wild-type rats were deeply anesthetized with sodium pentobarbital (40 mg/kg; Kyoritsu Seiyaku) and perfused with 0.05 M phosphate-buffered saline (PBS) followed by 4% paraformaldehyde in 0.05 M phosphate buffer (PB) for brain collection. The brain was immersed in the same fixative overnight at 4 °C, followed by 30% sucrose in 0.05 M PB at 4 °C until it sank. Coronal sections of the brain with 50-µm thickness were prepared with a cryostat and stored in the cryoprotectant solution at −30 °C until staining. Every fourth section throughout the ARC or every second section throughout the AVPV collected from 3 animals was used for the double in situ hybridization for *Kiss1* and *Htr2c*.

For double in situ hybridization for *Kiss1* and *Htr2c*, the hypothalamic cDNA of rats was synthesized, and the *Kiss1*-specific fluorescein isothiocyanate (FITC)-labeled cRNA probe (position 33-348, GenBank Accession No. AY196983) and *Htr2c*-specific digoxigenin (DIG)-labeled cRNA probe (position 52-1257, GenBank Accession No. NM_012765) were synthesized from the cDNA by using a FITC- or DIG-labeling kit (Roche Diagnostics) as previously described^74^. The brain sections were hybridized with 1 µg/mL anti-sense *Kiss1* and *Htr2c* cRNA probes at 60 °C overnight. Hybridized sections were incubated with a peroxidase (POD)-conjugated anti-FITC antibody (1:1000, Roche Diagnostics) and tyramide signal amplification (TSA) Plus FITC Kit (1:100; Perkin Elmer) to detect the *Kiss1* cRNA probe. After inactivation of POD by incubating the sections in 0.1 N hydrochloric acid for 15 min, the *Htr2c* cRNA probe was detected using the POD-conjugated anti-DIG antibody (1:1000; Roche Diagnostics), TSA Plus Biotin Kit (1:100; Perkin Elmer), and DyLight 594-conjugated streptavidin (1:500; Thermo Fisher Scientific). The brain sections were mounted on slide glasses, and fluorescence images were obtained on an ApoTome fluorescence microscope (Carl Zeiss). *Kiss1*- and *Htr2c*-expressing cells were unilaterally counted in both the ARC (from 1.8 to 4.2 mm posterior to the bregma; 12 sections in total for each brain) and AVPV (from 0.5 mm anterior to 0.5 mm posterior to the bregma; 10 sections in total for each brain) regions. The ratio of *Kiss1*-expressing cells coexpressing the *Htr2c* gene was calculated.

### Central administration of serotonin or 5HT2CR agonist/antagonist in female goats with MUA recordings in the ARC

OVX goats (n = 14) were stereotaxically implanted with an array of bilateral recording electrodes targeting the caudal ARC under halothane anesthesia, as described previously^75^. An 18-G stainless steel guide cannula was implanted in the LV for intracerebroventricular administration of serotonin hydrochloride (Sigma‒Aldrich), MK212 hydrochloride (a selective 5HT2CR agonist; R&D Systems), or SB206553 hydrochloride (5HT2B/2CR antagonist; Santa Cruz Biotechnology) as described previously^75^. Briefly, it is reported that MK212 showed selectivity for the 5HT2CR over the 5HT2AR and 5HT2CR^76^. SB206553 showed considerable selectivity over 5HT2A receptors but retained a significant affinity for the 5HT2BR^77^. The placement of the electrodes implanted in the ARC and the guide cannula implanted in the LV was verified with X-ray photographs. The goats showing MUA volleys in a prior screening were used in the experiments. Serotonin was dissolved in saline at 0.5 or 5 µmol/400 µL, and MK212 and SB206553 were dissolved in distilled water at 0.5 or 5 µmol/400 µL and 1 µmol/mL, respectively. The MUA was recorded in conscious animals, and a MUA volley was determined as the electrophysiological manifestation of GnRH pulse generator activity, as reported previously^78,79^. In addition, the goats were fitted with an 18-G jugular catheter (Medicut; Covidien Japan) at least 1 day before the blood sampling. The mean interval of MUA volleys (Tm) was determined by recording the MUA volleys for 5 h on the day before the experiment of serotonin or 5HT2CR agonist/antagonist treatment. Serotonin (0.5 or 5 µmol/400 µL/head) or MK212, a 5HT2CR agonist (0.5 or 5 µmol/400 µL/head), was infused at a 400 µL/min flow rate for 1 min into the LV at a half time of Tm after the MUA volley (Tm/2; see Fig. 4a for details). The LV infusion of SB206553, a 5HT2B/2CR antagonist (1 µmol/mL at 0.33 mL/min flow rate) by a syringe pump (Chemyx), was started after the second MUA volley was detected. The antagonist was continuously infused until the timing of serotonin injection at Tm/2 after the third MUA volley (see Fig. 4a for details). Blood samples were collected every 6 min for the 2-h MUA recording period. Plasma samples were obtained by immediate centrifugation and stored at −20 °C until assayed for LH.

### Assay for plasma LH and glucose concentration

Plasma LH concentrations in rats and goats were measured by double-antibody radioimmunoassay (RIA) as previously described^80,81^ using a rat LH RIA kit and an ovine LH RIA kit, respectively, provided by the National Hormone and Peptide Program (Harbor-UCLA Medical Center). The least detectable level of rat LH in 50-µL plasma samples was 0.156 ng/mL, and the intra- and interassay coefficients of variation were 8.3% and 9.7% at 0.476 ng/mL, respectively. The least detectable level of goat LH in 50-µL plasma samples was 0.098 ng/mL, and the intra- and interassay coefficients of variation were 6.1% at 1.16 ng/mL and 7.7% at 1.98 ng/mL, respectively.

Plasma glucose concentrations were measured by the glucose oxidase method using a commercial kit (Glucose CII-test; Wako Pure Chemical), as previously described^26^. The least detectable level in 1.5-µL plasma samples was 0.5 mg/mL. The intra- and interassay coefficients of variation were 5.5% at 1.35 mg/mL and 6.3% at 1.27 mg/mL, respectively.

### Statistical analysis

Statistical differences in the percentage of *Kiss1*-positive cells coexpressing *Htr2c* in the rostral, middle, and caudal parts of the ARC were determined by one-way ANOVA and that in the rostral and caudal parts of the AVPV were determined by Student’s *t-*test. LH pulses were identified with the PULSAR computer program^82^, as previously described^83,84^. Statistical differences in LH pulse parameters (the mean LH concentrations and the frequency and amplitude of LH pulses) and the AUC of plasma glucose levels among the xylose (iv)- and 2DG (iv)-treated rats administered vehicle (MBH) or fluoxetine (MBH) were determined by two-way (2DG and fluoxetine treatments as main effects) ANOVA followed by analyses of simple main effects. Statistical differences in plasma glucose levels during the last 2-h sampling period among the aforementioned groups were determined by three-way repeated measures (2DG and fluoxetine treatments, and time as main effects) ANOVA followed by analyses of simple-simple main effects. Statistical differences in the AUC of percent changes in MBH serotonin levels between xylose (DR)- and glucose (DR)-treated rats, mean LH concentrations, LH pulse frequencies, LH pulse amplitudes, and the AUC of plasma glucose levels between the 2DG (iv)-treated rats administered xylose (DR)- and glucose (DR) were determined by Student’s *t-*test. Statistical differences in percent change of MBH dialysate serotonin levels between xylose (DR)- and glucose (DR)-treated rats and plasma glucose levels during the last 2-h sampling period between the 2DG (iv)-treated rats administered xylose (DR) or glucose (DR) were determined by two-way repeated measures (glucose treatment and time as main effects) ANOVA. The percent change in mean LH concentration was calculated as the ratio of the mean LH levels during the Tm/2 after the drug administration to the mean during Tm/2 before the administration. Statistical differences in the mean MUA volley intervals, the percent change in mean LH levels between vehicle- and serotonin (0.5 or 5 µmol)-treated goats and between vehicle- and MK212 (0.5 or 5 µmol)-treated goats were determined by one-way ANOVA followed by Dunnett’s test. Statistical differences in the mean MUA volley intervals between the serotonin-injected goats with SB206553 or vehicle infusion were determined by two-way repeated measures (SB206553 treatment and time as main effects) ANOVA. Statistical differences in the percent change in mean LH levels between SB206553 or vehicle infusion groups were determined by Student’s *t*-test.

## Data Availability

The datasets generated during and/or analyzed during the current study are available from the corresponding authors on reasonable request.
